# Case report of laryngeal infection by *Clinostomum complanatum* 24 days after ingestion of raw fish

**DOI:** 10.1097/MD.0000000000034000

**Published:** 2023-06-02

**Authors:** Hyun-Gyu Kim, Ji-Hee Han, Ji-Yoon Kwak, Han Kyu Jeon, Sang-Soo Lee, Hyun Jin Kim, Seul Ki Song, Byoung-Kuk Na, Ra-Ri Cha

**Affiliations:** a Department of Internal Medicine, Gyeongsang National University School of Medicine, Jinju, Republic of Korea; b Department of Internal Medicine, Gyeongsang National University Changwon Hospital, Changwon, Republic of Korea; c Department of Otorhinolaryngology-Head and Neck Surgery, Gyeongsang National University School of Medicine, Gyeongsang National University Changwon Hospital, Changwon, South Korea; d Department of Parasitology and Tropical Medicine, Institute of Health Sciences, Gyeongsang National University College of Medicine, Jinju, Republic of Korea; e Department of Convergence Medical Science, Gyeongsang National University, Jinju, Republic of Korea.

**Keywords:** *Clinostomum complanatum*, human case, laryngopharyngitis

## Abstract

*Clinostomum complanatum* is a laryngeal fluke whose hosts include birds and mammals.^[1]^ In humans, infection occurs accidentally during the consumption of raw freshwater fish.^[1,2]^

**Patient concerns:**

A 59-year-old female presented to our hospital with throat pain and globus sensation. The patient had been prescribed Proton Pump Inibitor for 3 weeks at another hospital. The patient continued the medication, but the discomfort persisted, and she was admitted to our hospital for further examination. The patient had eaten raw fish 24 days before, and the symptoms occurred after eating the raw fish. Endoscopy under sedation showed a fluke, with an approximate length of 8.0 mm and width of 3.2 mm, on the interaryepiglottic fold, with active motility on the mucosa.

**Diagnosis interventions:**

It was extracted from the larynx using biopsy forceps and identified as *C complanatum.*

**Outcomes:**

After the fluke was removed, symptoms improved, and the patient was discharged. The globus symptoms completely resolved at the last follow-up visit.

**Lessons:**

To the best of our knowledge, this is an endoscopically diagnosed and treated case of human infection by *C complanatum* in Korea after the longest period of infection. This suggests that *C complanatum* can survive for up to 3 weeks or more in the gastrointestinal tract. Endoscopy is a useful tool for the diagnosis and treatment of patients with atypical extraesophageal symptoms who do not respond to Proton Pump Inibitors.

## 1. Introduction

*Clinostomum complanatum* is a laryngeal fluke whose hosts include birds and mammals.^[1]^ *C complanatum* usually has 2 intermediate hosts throughout its life cycle. Freshwater snails act as the first intermediate hosts, whereas freshwater fish and brackish fish serve as second intermediate hosts.^[1,2]^ *C complanatum* parasitizes the throat or esophagus of birds that mainly feed on fish and is transmitted through freshwater fish.^[3]^ In humans, infection occurs accidentally during the consumption of raw freshwater fish.^[1,4]^ If humans ingest freshwater fish, a secondary intermediate host infected with the parasite metacercaria, the larvae will break out in the stomach. After moving through the esophagus, it attaches to the mucous membrane of the pharynx, causing laryngitis.^[3,5]^ Here, we report a case of a 59-year-old female with laryngeal infection by *C complanatum* that was endoscopically diagnosed and treated.

## 2. Case report

A 59-year-old female with no underlying disease visited our hospital with throat pain and globus sensation. The patient had been prescribed PPI (Proton Pump Inhibitor) for 3 weeks at another hospital. The patient continued the medication, but the discomfort persisted, and she was admitted to our hospital for further examination. History taking was repeated for atypical globus symptoms since the patient did not respond to PPI. The patient had eaten raw fish 24 days before, and the symptoms occurred after eating the raw fish.

**Figure 1.**
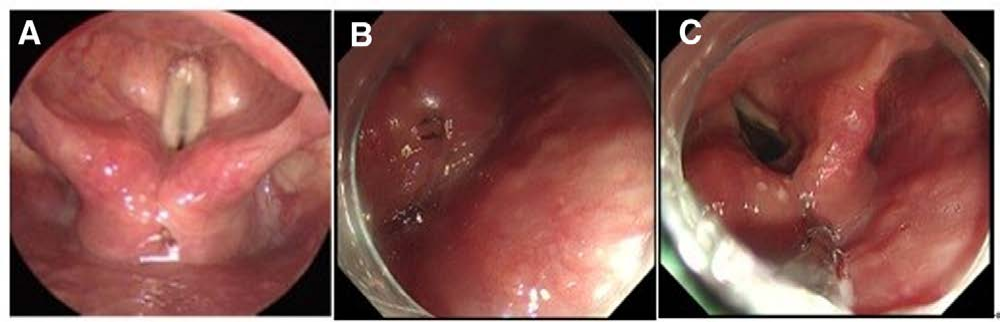
Photographs of *Clinostomum complanatum*. Endoscopy shows a parasite on the surface of the interarytenoid mucosa.

Endoscopy was performed under sedation and showed a fluke, with an approximate length of 8.0 mm and width of 3.2 mm, on the interaryepiglottic fold, with active motility on the mucosa. It was extracted from the larynx using biopsy forceps. The obtained specimen was sent for parasitological consultation and identified the organism as an adult parasite worm, *C complanatum* (Fig. 1).

**Figure 2.**
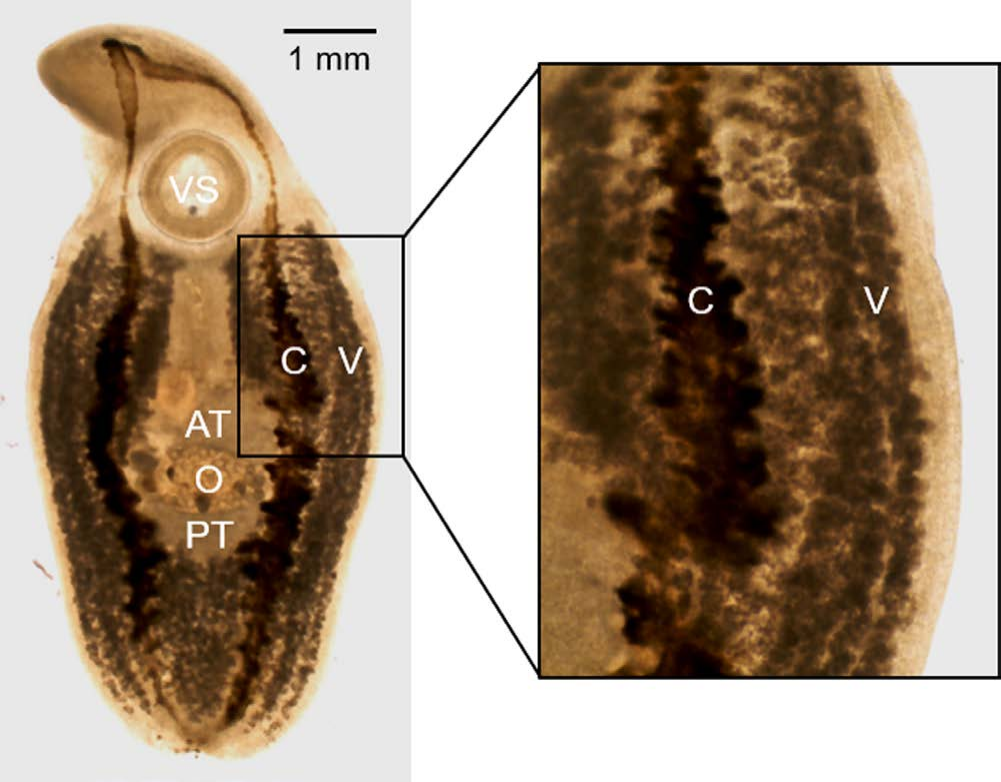
Light microscopic image of *Clinostomum complanatum*. AT = anterior testis, C = cecum (smooth folded lumen), O = ovary, PT = posterior testis, V = vitellaria, VS = ventral sucker.

Morphological examination of the *C complanatum* under light microscopy measured 8 mm in length and 3 mm in width. The ventral sucker, anterior testis, ovary, posterior testis, cecum, and vitellaria were identified in the removed parasites, and the laryngeal fluke was identified to be *C complanatum* (Fig. 2).

No bleeding or mucosal damage was observed after the removal. No hospitalization was required. After the fluke was removed, symptoms improved, and the patient was discharged. The globus symptoms completely resolved at the last follow-up visit.

## 3. Discussion

In Korea, human infection with *C complanatum* was first reported by the Society of Parasitology in 1995, and since then, 7 cases of human infection have been reported (Table 1). *C complanatum* was removed using a laryngoscope in 3 cases, using endoscopy in 3 cases, and via surgery in 1 case.^[3,6–11]^

**Table 1:** Cases of *Clinostomum complanatum* infections in Korea.

No.	Study	Age (yr)	Sex	Site	Causative food	Removal methods	Duration (d)	Division	Symptom
1	Chung et al (1995)^[6]^	56	M	Pharynx	Raw fresh-water fish	Removal under laryngoscope	3–4	Otolaryngology	Throat pain
2	Park et al (2009)^[7]^	33	M	Larynx (arytenoid region)	Raw fresh-water fish (perch)	Removal under drug-induced sleep flexible endoscopy	7	Internal medicine	Throat pain
3	Jung et al (2015)^[3]^	13	F	Pharynx	Raw fresh-water fish (mullet)	Surgery	4	Otolaryngology	Globus sensation
4	Lee et al (2017)^[8]^	46	F	Larynx (left aryepiglottic fold)	Raw fresh-water fish (raw perch and mullet)	Removal under drug-induced sleep flexible endoscopy	7	Internal medicine	Globus sensation Throat pain
5	Song et al (2018)^[9]^	20	M	Larynx (left arytenoid)	Raw fresh-water fish	Removal under laryngoscope	2	Otolaryngology	Globus sensation
6	Kim et al (2019)^[10]^	46	F	Pharynx	Raw fresh-water fish	Removal under drug-induced sleep flexible endoscopy	5	Otolaryngology	Globus sensation
7	Moon and Park (2020)^[11]^	22	F	Larynx (left aryepiglottic fold)	Raw fresh-water fish	Removal under laryngoscope	3	Otolaryngology	Globus sensation Throat pain

In patients with *C complanatum* infection reported to date, symptoms (globus sensation, throat pain) appeared within 2 days at the earliest and within 7 days at the latest, after eating raw fish. In our patient, symptoms appeared 2 days after consuming the raw fish, also. However, the patient was diagnosed with globus symptoms at another hospital and underwent PPI treatment, and endoscopy was performed after 3 weeks at our hospital. Endoscopy confirmed laryngitis caused by a *C complanatum* fluke. This suggests that *C complanatum* can survive for up to 3 weeks or more in the gastrointestinal tract. Moreover, it can travel through the esophagus and into the mucous membrane of the pharynx for up to 3 weeks. To the best of our knowledge, this is an endoscopically diagnosed and treated case of human infection by *C complanatum* in Korea after the longest period of infection. The type and duration of food intake are important when considering medical history. Even if a certain period has passed since raw fish consumption, if a history of raw fish consumption and symptoms such as sore throat are present, infection by *C complanatum* should be considered. Therefore, active history-taking and workups are important.

The only well-known treatment for *C complanatum* infection is the elimination of the fluke. In this case, the fluke was successfully removed under sedation using an endoscope. The endoscope was used to ensure that no flukes remained in the larynx, pharynx, esophagus, or stomach.

*Clinostomum complanatum*, a new parasitic disease, was first reported in Korea in 1995, and the number of cases has been gradually increasing. This phenomenon is due to the fact that various environmental changes appear due to global warming, and these changes increase the chance of microbial infection in fish, shellfish, meat and thus increase the chance of human infection through food.^[12]^ Therefore, it is expected that the occurrence and detection rate of *C complanatum* will gradually increase due to the continuation of eating raw fish and the acceleration of global warming. Therefore, we need to be aware of these diseases.

In conclusion, although laryngopharyngitis caused by *C complanatum* is rare, the number of reports on this condition is increasing. In this case, detailed history taking and active endoscopy were required when a globus patient presented with atypical symptoms and did not respond to PPI. If a patient has a history of consuming raw fish, active endoscopy is helpful for the diagnosis of parasitic infections.

## Author contributions

**Conceptualization:** Hyun-Gyu Kim, Ji-Hee Han, Ji-Yoon Kwak.

**Methodology:** Han Kyu Jeon, Sang-Soo Lee, Seul Ki Song, Byong-Kuk Na.

**Visualization:** Hyun Jin Kim.

**Writing – original draft:** Hyun-Gyu Kim.

**Writing – review & editing:** Ra ri Cha.
